# Downregulation of LncRNA SNHG7 Sensitizes Colorectal Cancer Cells to Resist Anlotinib by Regulating miR-181a-5p/GATA6

**DOI:** 10.1155/2023/6973723

**Published:** 2023-01-14

**Authors:** Deng Pan, Kehe Chen, Ping Chen, Yu Liu, Yingying Wu, Jingning Huang

**Affiliations:** ^1^The First Affiliated Hospital of Jinan University, Guangzhou 510632, China; ^2^Department of Medical Oncology, Guangxi Academy of Medical Sciences and the People's Hospital of Guangxi Zhuang Region, Nanning, Guangxi 530021, China; ^3^Department of Oncology, Chengdu Seventh People's Hospital, Chengdu Tumor Hospital, Chengdu, Sichuan 610041, China

## Abstract

Long noncoding RNAs are a novel class of regulators in human cancers. It has been reported that small nucleolar RNA hostgene 7 (SNHG7) can sponge microRNAs to regulate colorectal cancer (CRC) progression. Given its important regulatory role in cancer biology, we wondered whether SNHG7 is involved in drug resistance to anlotinib (ATB) in CRC. To answer this, we quantified the expression of SNHG7 by quantitative real-time PCR. We performed the Cell Counting Kit-8 and Colony formation assay, flow cytometric analysis, RNA pull-down, RNA-binding protein immunoprecipitation assay, and Luciferase reporter assay to confirm the interaction among SNHG7, miR-181a-5p, and GATA6. We found that SNHG7 was significantly upregulated in CRC tissues and cell lines and ATB-resistant cell lines, which was closely related to the poor overall survival of patients. Loss-of-function studies demonstrated that SNHG7 knockdown can inhibit CRC cell proliferation, increase apoptosis, and sensitize CRC cells to resist ATB. Mechanistic studies showed that SNHG7 acted as a competitive endogenous RNA to sponge miR-181a-5p to regulate the expression of GATA6, thereby promoting ATB resistance in ATB-resistant cell lines. In conclusion, SNHG7 plays an important role in ATB resistance, and it may be used to monitor ATB resistance in CRC.

## 1. Introduction

Colorectal cancer (CRC) is the gastrointestinal tumor with the third highest incidence and the second mortality worldwide [1, 2]. With the advent of modern medicine, more and more chemotherapy drugs and novel targeted therapies have been introduced into clinical practice, improving patient prognosis and overall survival (OS) [3, 4]. However, resistance to chemotherapy drugs has become an intractable threat to survival in patients with CRC [5]. Therefore, studying the underlying mechanisms of drug resistance in CRC is of great significance for clinical treatment.

A variety of tumors including long noncoding RNAs (lncRNAs), microRNAs (miRNAs), and functional genes (mRNA) cooperatively form a complex regulatory network (lncRNA–miRNA–mRNA) to regulate the proliferation, apoptosis, and drug resistance of tumor cells [6–8]. LncRNAs are a group of noncoding RNAs with more than 200 nucleotides in length [9]. SNHG7 is a lncRNA containing 2157 nucleotide acids [10]. It is mainly localized in the cytoplasm, acting as a competitive endogenous RNA (ceRNA) to sponge miRNAs to regulate the expression of target genes [11]. For example, SNHG7 is highly expressed and regulates the gene expression in multiple human malignancies, including miR-193b/FAIM2 in lung cancer [12], miR-503/cyclin D1 in prostate cancer [13], and miR-34a/Notch-1 in breast cancer [14]. Interestingly, Shan et al. [11] and Li et al. [15] independently reported that SNHG7 expression was upregulated in CRC to regulate miR-216b/GALNT1 and miR-34a/GALNT7 to promote cell proliferation, migration, and invasion in CRC. Given that, we surmise that SNHG7 expression was significantly related to poor disease-free survival and OS of patients with CRC for whom drug resistance to anlotinib (ATB) is commonplace. However, there is a paucity of research into the role of SNHG7 in the drug resistance of CRC.

MiR-181a-5p is a multifaceted miRNA involved in the regulation of many diseases. For example, miR-181a-5p was found to be upregulated in pancreatic cancer (PC) and breast cancer [16, 17] while down-regulated in type 2 diabetes mellitus [18], cerebral ischemic injury [19], and prostate cancer [20]. Interestingly, the downregulation of miR-181a-5p in CRC has been detected. For instance, miR-181a-5p was downregulated by lncRNA CRNDE through sponging to modulate the activity of Wnt/*β*-catenin signaling, thereby regulating the progression and chemoresistance of CRC [21]. Moreover, miR-181a-5p was sponged by circNSUN2, which increases the expression of ROCK2 and promotes the progression of CRC [22]. Therefore, it is possible that miR-181a-5p is involved in ATB resistance in CRC.

GATA6 is a member of GATA family and is located throughout the gastrointestinal epithelium with a particularly high level in the proliferative crypt compartment. Deletion of GATA6 in the intestine results in impaired crypt cell proliferation, crypt-to-surface epithelial migration, lineage maturation, and gene expression in the mature mouse colonic epithelium [23, 24]. GATA6 is a target gene of miRNAs and can regulate cancer progression. In colon cancer, GATA6 overexpression regulates the expression of the urokinase-type plasminogen activator (uPA) gene, which contributes to colorectal tumorigenesis and tumor invasion [25]. In human colon cancer, GATA6 upregulation decreases the expression level of 15-LOX-1 [26]. Considering its regulator role in gene expression, we speculate that GATA6 may contribute to the resistance to ATB.

ATB, a highly selective multi-targeted tyrosine kinase inhibitor, has been widely used in chemotherapy of non-small-cell lung cancer, soft tissue sarcoma, renal carcinoma, hepatocarcinoma, and gastric cancer [27, 28]. ATB suppresses angiogenesis, metastasis, cell proliferation, and multidrug resistance in CRC [29]. ATB can effectively inhibit the activity of VEGFR, PDGFR, FGFR, c-kit, and other kinases, thus suppressing tumor angiogenesis and growth. As a potential third-line therapy, ATB is safe and effective for treating patients with metastatic CRC [30]. ATB resistance in lung cancer has become a limiting factor of clinical efficacy [31]. Considering the wide prescription of ATB in CRC treatment, understanding the underlying mechanisms of ATB resistance in CRC would be conducive to improving the efficacy of clinical therapy involving ATB. In this study, we found that SNHG7 was upregulated in CRC, which exhibited a reverse correlation with prognosis. Moreover, we demonstrated that SNHG7 might act as a ceRNA to sponge miR-181a-5p to upregulate the expression of GATA6, contributing to ATB resistance in CRC cells.

## 2. Materials and Methods

### 2.1. Cell Culture

Human CRC cell lines, including HCT116, HT29, HCT8, LOVO, SW480, SW620, and DLD1, and the human epithelial cell line NCM460, were obtained from the American Type Culture Collection (Manassas, VA, USA). Cells were cultured in RPMI-1640 (Thermo Fisher Scientific, Waltham, MA, USA) containing 10% fetal bovine serum (FBS, Thermo Fisher Scientific), 100 *μ*g/mL of streptomycin (Invitrogen, Carlsbad, CA, USA), and 100 U/mL of penicillin (Invitrogen) in a humidified incubator at 37°C with 5% CO_2_. The cells with 60% confluence were used in subsequent experiments.

### 2.2. Tissue Samples

A total of 50 patients with CRC (27 males and 23 females) aged 39–67 years were recruited from June 2019 to July 2021 at the People's Hospital of Guangxi, Nanning, China. None of the patients underwent chemoradiotherapy and anticancer treatments before surgery. All CRC tissues and adjacent normal tissues (5 cm away from the tumor foci) were collected during surgery, and CRC diagnosis was verified by two pathologists independently. The tissue specimens were refrigerated in liquid nitrogen immediately and stored at −80°C. All patients provided written informed consent. The present study was approved by the People's Hospital of Guangxi Institutional Review Board (approval no. GXNN-KY-2175).

### 2.3. Construction of the ATB-Resistant CRC Cells

HCT116 and LOVO cells (1 × 10^7^) were exposed to 100 mg/mL *N*-ethyl-*N*-nitrosourea (Sigma-Aldrich, Burlington, MA, USA) for 24 hours, followed by treatment with a gradient concentration of ATB to induce ATB resistance. During the first five days, HCT116 and LOVO cells were exposed to ATB (4 *μ*g/mL), and the medium was changed every day. In the next two months, 6, 8, 10, and 12 *μ*g/mL of ATB were used to treat the cells [32]. Finally, about 100 variable cells exposed to ATB (12 *μ*g/mL) were recovered. After proliferation for about one month, the ATB-resistant HCT116 and LOVO cells, named HCT116/ATB and LOVO/ATB separately, were used for functional assays.

### 2.4. Cell Transfection

The siRNAs targeting SNHG7, miR-181a-5p mimic, and miR-181a-5p inhibitor were designed by Shanghai GeneChem Co., Ltd., Shanghai, China. The overexpression constructs, oe-SNHG7 and oe-GATA6, were prepared by Shanghai GenePharma Co., Ltd, Shanghai, China, by cloning the open reading frame of *GATA6* into the expression vector pcDNA 3.1(+) (Sigma-Aldrich; Merck KGaA, Darmstadt, Germany). An empty vector [(pcDNA3.1(+)] was used as a negative control for GATA6 overexpression. A total of 20 nM of each construct was transfected into cells at 37°C for 15 minutes using Lipofectamine 3000 (Thermo Fisher Scientific) following the manufacturer's protocol. After 48 hours, the transfected cells were harvested to perform subsequent experiments. The sequences were as follows:

si-NC: 5′-TTCTCCGAACGTGTCACGT-3′;

si-SNHG7: 5′-UUAGCAGAGUAAUUUGCACUU-3′;

mimics-NC: 5′-TTCTCCGAACGTGTCACGT-3′;

miR-181a-5p mimics: 5′-AACAUUCAACGCUGUCGGUGAGU-3′;

inhibitor-NC: 5′-UUCUCCGAACGUGUCACGUTT-3′;

miR-181a-5p inhibitor: 5′-ACUCACCGACAGCGUUGAAUGUU-3′.

### 2.5. RNA Extraction and Quantitative Real-Time PCR (qRT-PCR)

Total RNA was extracted using TRIzol reagent (Thermo Fisher Scientific) and was evaluated and quantified using a NanoDrop ND2000 microspectrophotometer (Thermo Fisher Scientific). Subsequently, the total RNA was reversely transcribed into cDNA according to the protocol of the Reverse Transcription System (Promega, Madison, WI, USA). qRT-PCR analysis was performed using the SYBR Green Master Mix (CWBIO, Beijing, China). The primers used in this study were synthesized by Sangon Biotech, Shanghai, China. The expression levels of relevant genes were determined by the 2^−*ΔΔ*Ct^ method. Glyceraldehyde-3-phosphate dehydrogenase (GAPDH) and U6 were used as internal reference genes [33]. Experiments were performed in triplicate. The primers used for qRT-PCR were:

SNHG7:

Forward: 5′-GTGACTTCGCCTGTGATGGA-3′,

Reverse: 5′-GGC CTCTATCTGTACCTTTATTCC-3′;

miR-181a-5p:

Forward: 5′-GGGCAGCCTTAAGAGGA-3′,

Reverse: 5′-GGC CTCTATCTGTACCTTTATTCC-3′;

GATA6:

Forward: 5′-AGCAAGATGAACGGCCTCAG-3′,

Reverse: 5′-GTTGTGGTGTGACAGTTGGC-3′;

GAPDH:

Forward: 5′-TCCTCTGACTTCAACAGCGACAC-3′,

Reverse: 5′-CACCCTGTTGCTGTAGCCAAATTC -3′;

U6:

Forward: 5′-CTCGCTTCGGCAGCACA-3′,

Reverse: 5′-AACGCTTCACGAATTTGCGT-3′.

### 2.6. Cell Counting Kit-8 (CCK-8) Assay

The CCK-8 assay was used to detect the proliferation of the HCT116/ATB and LOVO/ATB cells. Briefly, 100 *μ*L of cell suspension was inoculated in a 96-well plate (1 × 10^3^ cells/well) and was cultured at 37°C, 5% CO_2_. After that, 10 *μ*L of CCK-8 solution (Sigma-Aldrich) was added to the cell culture at 0, 24, 48, and 72 hours. Finally, the optical density value was recorded at 450 nm using a Microplate reader (BioTek, Winooski, VT, USA).

To analyze the cytotoxicity of ATB to HCT116/ATB and LOVO/ATB cells, different concentrations of ATB were added to the cell culture. Then, the plate was cultured for 48 hours at 37°C, 5% CO_2_. Then, 10 *μ*L of CCK8 solution was added to each well, and the cells were further cultured for 1–4 hours. The cytotoxicity of ATB to HCT116/ATB and LOVO/ATB cells was measured using a Microplate reader (BioTek).

### 2.7. Colony Formation Assay

HCT116/ATB and LOVO/ATB cells (1 × 10^3^) at the logarithmic growth stage with diverse treatments were inoculated in dishes containing the Dulbecco's Modified Eagle Medium (DMEM) medium containing 10% FBS. The cells were cultured at 37°C for 2–3 weeks until clones formed. The clones were fixed with 4% paraformaldehyde (Sigma-Aldrich) and stained with GIMSA solution (Sigma-Aldrich) for 10–30 minutes at room temperature. The colonies were analyzed using ImageJ software v1.8.0 (National Institutes of Health, USA). Clone formation rate was evaluated by the formula: “clone formation rate = numbers of clones/numbers of cells × 100%.”

### 2.8. Luciferase Reporter Assay

To analyze the regulation of SNHG7 by miR-181a-5p, the reporter plasmid was constructed by fusing SNHG7 cDNA into the 3'-UTR region of the Luciferase gene. To analyze the regulation of GATA6 miR-181a-5p, the reporter plasmid was constructed by fusing GATA6 CDS with the CDS of the Luciferase gene. The synthesized miR-181a-5p and reporter plasmid were co-transfected into HCT116/ATB and LOVO/ATB cells. The luciferase activities were measured using the Luciferase Assay System (Promega) to reflect the degradation of Luciferase: SNHG7 or Luciferase: GATA6.

### 2.9. RNA Pull-Down

The PCR products were verified by gel electrophoresis and purified by phenol:chloroform extraction. Biotin-labeled miR-181a-5p sense and antisense hybridization probes were obtained by *in vitro* transcription (Roche, Basel, Switzerland). The probes were incubated with cytoplasmic extracts from HCT116/ATB or LOVO/ATB cells to form miRNA–lncRNA compounds, which were separated using magnetic beads (Thermo Fisher Scientific) labeled with avidin. Finally, the qRT-PCR analysis determined the enrichment of SNHG7 in the immunoprecipitated RNA.

### 2.10. RNA Binding Protein Immunoprecipitation (RIP) Assay

RIP was performed to confirm the interaction between SNHG7 and miR-181a-5p using the EZMagna RIP Kit (Millipore, Burlington, MA, USA). Briefly, HCT116/ATB and LOVO/ATB cells were lysed in a complete RIP lysis buffer. Then, cell lysates were incubated with the RIP buffer containing magnetic beads coated with anti-Ago2 or anti-IgG antibodies for 4 hours at 4°C. Finally, qRT-PCR was performed to determine the enrichment of SNHG7 and miR-181a-5p in the immunoprecipitated RNA.

### 2.11. Flow Cytometric Analysis (FITC–PI)

HCT116/ATB and LOVO/ATB cells (1.5 × 10^5^) were cultured in six-well plates and then harvested in centrifuge tubes. The adherent cells were treated with trypsin–Ethylene Diamine Tetraacetic Acid (EDTA) solution (J&K Scientific, Beijing, China) and were transferred to new centrifuge tubes. The cells were collected by centrifugation at 200*g* for 5 minutes, followed by one wash with fetal bovine serum (PBS). Then, the cells were resuspended in 195 *μ*L of binding buffer and incubated with 10 *μ*g/mL Annexin V-fluorescein isothiocyanate (FITC; Sigma-Aldrich) at room temperature for 10 minutes in dark. The cells were collected by centrifugation at 200*g* for 5 minutes, resuspended in 190 *μ*L of binding buffer, and kept on ice. Then, 10 *μ*L of propidium iodide (PI, Sigma-Aldrich) was added to the binding buffer. Apoptosis of HCT116/ATB and LOVO/ATB cells was analyzed using an FACScan flow cytometer (Becton Dickinson, Franklin Lakes, NJ, USA).

### 2.12. Western Blot Analysis

HCT116/ATB and LOVO/ATB cells were washed with PBS buffer. Then, the cells were resuspended and lysed using radioimmunoprecipitation assay lysis buffer (Thermo Fisher Scientific) to collect total protein. The concentration of proteins was measured by the bicinchoninic acid method. In total, 50 *μ*g of protein was separated on a 12.5% sodium dodecyl sulfate-polyacrylamide-polyacrylamide gel electrophoresis and transferred to a polyvinylidene fluoride (PVDF) membrane (Sigma-Aldrich). After blocking by 1% bovine serum albumin (BSA) for 1 hour, the PVDF membranes were incubated with the primary specific antibody of Bcl-2 (1 : 1,000; CST), caspase-3 (1 : 1,000; CST), GATA6 (1 : 1,000; CST), and GAPDH (1 : 1,000; CST) at 4°C for 24 hours. Then, the membranes were incubated with corresponding HRP-labeled Goat Anti-Rabbit IgG (H+L) (Abcam, Cambridge, UK). Finally, the PVDF membrane was stained using NBT/BCIP Reagent Kit (Thermo Fisher Scientific) and quantified using ImageJ software. GAPDH was used as an internal control. The experiment was done with three biological triplicates.

### 2.13. Animal Models Establishment

Male BALB/c nude mice (*n* = 16, 4–5 weeks old, 15–25 g) were purchased from the National Laboratory Animal Center (Beijing, China) and raised in specific pathogen-free conditions with regulated day–night cycles. A total of 1 × 10^7^ transfected HCT116/ATB cells were subcutaneously injected into the flanks of nude mice. Tumor size was measured by a bilateral calliper every 7 days until 28 days post injection. Tumor volume calculations were based on the formula (length × width 2/2). Subsequently, the mice were sacrificed by cervical dislocation under anesthesia. Then, the tumor volume was determined. All experiments abided by the Institutional Animal Care of the People's Hospital of Guangxi, China, and were approved by the Experimental Animal Ethics Committee of the People's Hospital of Guangxi, China (approval no. IR-D-2022-2-18).

### 2.14. Statistical Analysis

The data from three biological replicates were analyzed and graphed by the GraphPad Prism software. All numerical data were presented as the means ± standard deviation (SD). Student's *t*-test and one-way analysis of variance followed by Tukey's *post hoc* test were used to analyze two and multiple groups, respectively. The correlations between the expression of SNHG7, miR-181a-5p, and GATA6 were determined by Spearman correlation analysis. OS rates were calculated using the Kaplan–Meier method with the log-rank test. *P* < 0.05 was considered as statistical significance.

## 3. Results

### 3.1. SNHG7 Is Upregulated in CRC Tissues and ATB-Resistant Cell Lines

**Figure 1:**
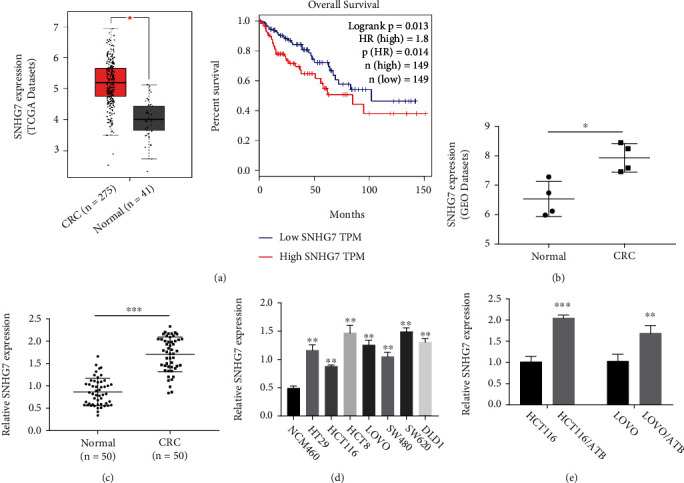
The expression of SNHG7 is upregulated in CRC. (a) Gene expression profiling interactive analysis (GEPIA) of the TCGA data to assess SNHG7 expression in rectal cancer (red column). (b) SNHG7 expression in the GEO dataset GSE75970. (c) qRT-PCR measurement to check the expression level of SNHG7 in 50 pairs of CRCs and the paracancerous tissues. (d) qRT-PCR analysis to determine the expression level of SNHG7 in CRC cell lines, HT29, HCT116, HCT8, LOVO, SW480, SW620, and DLD1 as well as in the normal cell line NCM460. (e) qRT-PCR analysis to detect SNHG7 expression in ATB-resistant cell lines HCT116/ATB and LOVO/ATB. The statistical significance of differences was evaluated by Student's *t*-test (∗*P* < 0.05, ∗∗*P* < 0.01, and ∗∗∗*P* < 0.001).

First, we checked the expression of SNHG7 in the gene expression data downloaded from the Cancer Genome Atlas (TCGA) and the Gene Expression Omnibus (GEO) websites (http://www.ncbi.nlm.nih.gov/geo/). SNHG7 expression was higher in CRC than in normal tissues (Figures 1(a) and 1(b)). Then, we analyzed the correlation of SNHG7 expression with the prognosis of CRC patients by Kaplan–Meier analysis. The OS of the patients with the higher expression of SHNG7 was shorter than that of the patients with the lower expression (Figure 1(a)). Then, we performed qRT-PCR to quantify SHNG7 transcripts in 50 pairs of CRCs and the paracancerous tissues. SHNG7 expression was significantly higher in the CRC tissues than in the healthy tissues (Figure 1(c)). In addition, the expression of SHNG7 inHT29, HCT116, HCT8, LOVO, SW480, SW620, and DLD1 CRC cells (Figure 1(d)) was higher than that in the control. Moreover, we checked the expression of SHNG7 in the ATB-resistant cell lines, HCT116/ATB and LOVO/ATB. The expression of SHNG7 in the two ATB-resistant cell lines was higher than that in the control (Figure 1(e)). These results indicate that SNHG7 expression is upregulated in CRC, which may contribute to the resistance to ATB.

### 3.2. Knockdown of SNHG7 Suppresses CRC Cell Proliferation and ATB Resistance *In Vitro* and *In Vivo*

**Figure 2:**
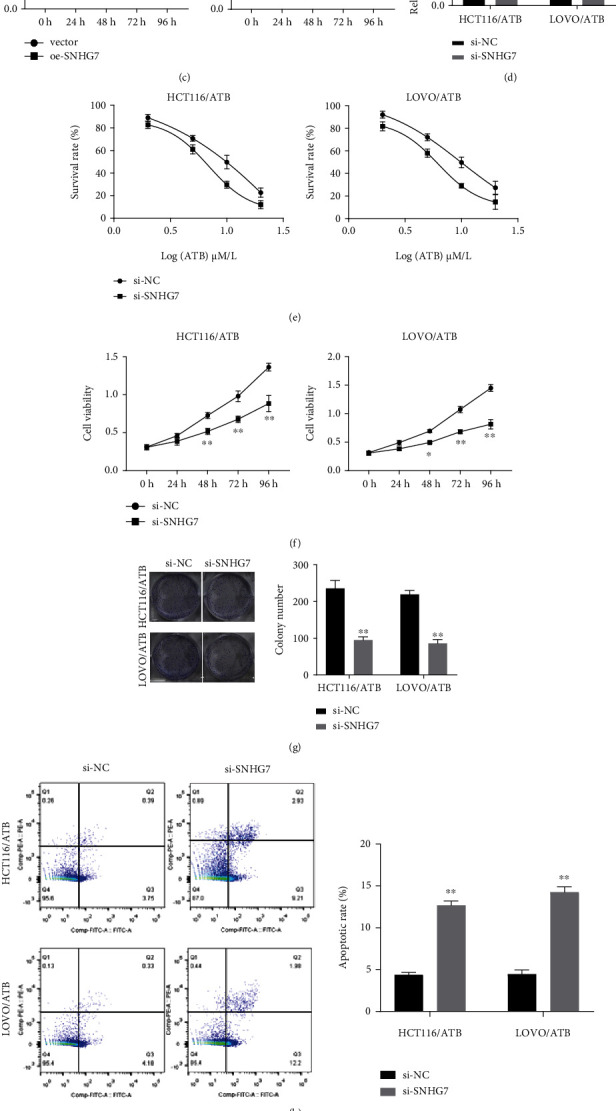
Knockdown of SNHG7 reverses ATB resistance in CRC cells. (a) qRT-PCR revealing the upregulation of SNHG7 in oe-SNHG7 cell lines (HCT116/ATB-oeSNHG7 and LOVO/ATB-oeSNHG7 cell lines). (b) Survival rate of the SNHG7-overexpressing HCT116/ATB and LOVO/ATB cell lines treated with 0, 2, 5, 10, and 20 *μ*M ATB. (c) CCK8 assay examining the cell viability of oeSNHG7 cell lines. (d) qRT-PCR measurement to check the expression of SNHG7 in siSNHG7 cell lines (HCT116/ATB-siSNHG7 and LOVO/ATB-siSNHG7 knockdown cell lines). (e) Survival rate of HCT116/ATB and LOVO/ATB cell lines with SNHG7 knockdown that are treated with 0, 2, 5, 10, and 20 *μ*M ATB. (f) CCK8 assay showing the decreased cell viability of siSNHG7 cell lines. (g) Clone-forming ability of HCT116/ATB and LOVO/ATB cell lines with SNHG7 knockdown. (h) Flow cytometric analysis detecting the apoptosis level of siSNHG7 cell lines and the control. Down-regulation of SNHG7 in HCT116/ATB and LOVO/ATB cell lines increases cell apoptosis. (i) Western blot determining the protein level of caspase-3 and Bcl-2. The expression level of caspase-3 was increased, whereas Bcl-2 was decreased in siSNHG7 cell lines. The statistical differences were evaluated by Student's *t*-test (∗*P* < 0.05, ∗∗*P* < 0.01, and ∗∗∗*P* < 0.001).

We speculated that SNHG7 overexpression would increase the resistance of CRC cells to ATB. To verify this, we first overexpressed SNHG7 in CRC cells.  Figure 2(a) reports the increased expression level of SNHG7 in the cells transfected with oeSNHG7. Then, we used the CCK-8 assay to evaluate the susceptibility of oe-SNHG7 cell lines to ATB. The results showed that SNHG7 overexpression significantly increased the survival rate and IC50 of the cells treated with different concentrations of ATB (Figure 2(b)). Given the importance of anti-apoptosis ability of tumors in developing drug resistance in many tumors, we evaluated the cell proliferation ability and apoptosis in ATB-resistant cell lines [34, 35]. Compared with the control, the cell viability of oe-SNHG7 cell lines was significantly increased (Figure 2(c)). Next, we set out to study the function of SNHG7 by loss-of-function analysis. We silenced the expression of SNHG7 in HCT116/ATB and LOVO/ATB cell lines to further study its role in resistance to ATB. As shown in Figure 2(d), the expression level of SNHG7 in si-SNHG7 cell lines was decreased by about 50%. The CCK-8 results showed that SNHG7 knockdown significantly decreased the survival rate and IC50 of the ATB-treated cells (Figure 2(e)). Compared with the control, the cell viability of si-SNHG7 cell lines was significantly decreased (Figure 2(f)). Colony formation assay showed that si-SNHG7 cell lines formed fewer clones than did the control (Figure 2(g)). These results indicated that si-SNHG7 knockdown significantly decreased cell proliferation ability in ATB-resistant cell lines. By contrast, the cell apoptosis rate was significantly higher than that in the control (Figure 2(h)). Both caspase-3 and Bcl-2 are apoptosis-related proteins. It was reported that caspase-3 can induce apoptosis in insect Sf9 cells, which can be blocked by Bcl-2 [36]. We determined the levels of these two proteins in si-SNHG7 cell lines using western blot. The results showed that the level of caspase-3 was increased, whereas the level of Bcl-2 was decreased in si-SNHG7 cell lines (Figure 2(i)), which may account for the anti-apoptosis ability of ATB-resistant cell lines. To study the role of SNHG7 in tumor growth, HCT116/ATB cells transfected with si-SNHG7 were injected into the mice to establish a xenograft tumor model. The tumor derived from the cells with SNHG7 knockdown exhibited a significantly smaller size (Figure S1A). Collectively, these results demonstrated that SNHG7 knockdown suppresses CRC cell proliferation and ATB resistance *in vitro* and *in vivo*.

### 3.3. SNHG7 Sponges miR-181a-5p in CRC

**Figure 3:**
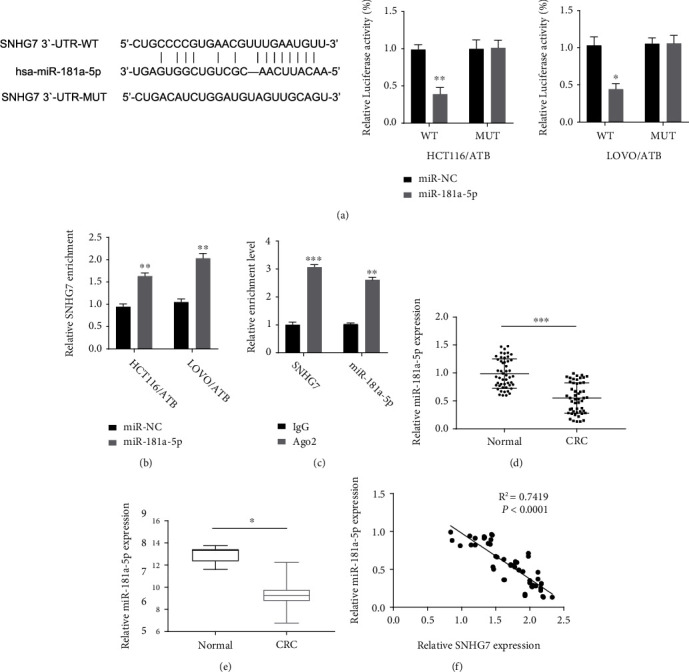
MiR-181a-5p is directly regulated by SNHG7. (a) Potential binding site of miR-181a-5p in SNHG7 predicted by StartBase3 and confirmed by Luciferase reporter assay in HCT116/ATB and LOVO/ATB cell lines. Overexpression of miR-181a-5p could inhibit Luciferase activity, but the inhibition was abolished when the potential site was mutated. (b) RNA pull-down assay showing that SNHG7 fragments were enriched effectively using the miR-181a-5p probe in HCT116/ATB and LOVO/ATB cell lines. (c) RIP-qRT-PCR showing SNHG7 and miR-181a-5p fragments were enriched by Ago2 in HCT116/ATB and LOVO/ATB cell lines. (d) qRT-PCR analysis of the expression of miR-181a-5p in 50 pairs of CRCs and the adjacent tissues. (e) StartBase3 analysis of the expression of miR-181a-5p in the data from TCGA. (f) Spearman correlation analysis revealing a significant negative correlation between SNHG7 and miR-181a-5p in 50 CRC cases. The statistical differences were evaluated by Student's *t*-test (∗*P* < 0.05, ∗∗*P* < 0.01, and ∗∗∗*P* < 0.001).

It has been reported that SNHG7 is localized to the cell cytoplasm and functions as a ceRNA to sponge miRNA to regulate gene expressions [11]. Therefore, we predicted the miRNA regulated by SNHG7. Startbase3 analysis revealed a potential binding site of miR-181a-5p in SNHG7 (Figure 3(a)). Then, we implemented the Luciferase reporter assay, RNA pull-down, and RIP to study the interaction between miR-181a-5p and SNHG7. Overexpression of miR-181a-5p inhibited luciferase activity in HCT116/ATB and LOVO/ATB cell lines; an effect was abolished when the potential miR-181a-5p binding site in SNHG7 was mutated (Figure 3(a)). RNA pull-down results showed that more SNHG7 fragments were enriched by the miR-181a-5p probe than by the control probe (Figure 3(b)). RIP results showed that SNHG7 and miR-181a-5p fragments were both immunoprecipitated using magnetic beads coated with anti-Ago2 but not with anti-IgG antibodies (Figure 3(c)). As SNHG7 was upregulated in CRC tissues (Figure 1), we anticipated that miRNA-181a-5p should be downregulated in CRC tissues. Indeed, we detected that the expression level of miR-181a-5p was decreased in the 50 CRC tissues relative to the adjacent tissues (Figure 3(d)). The TCGA data also revealed the same expression pattern (Figure 3(e)). Moreover, the Spearman correlation analysis showed a significant negative correlation between the expression of SNHG7 and miR-181a-5p (Figure 3(f)). Together, these results demonstrate that miR-181a-5p is sponged and regulated by SNHG7.

### 3.4. SNHG7 Regulates GATA6 Expression via Sponging miR-181a-5p

**Figure 4:**
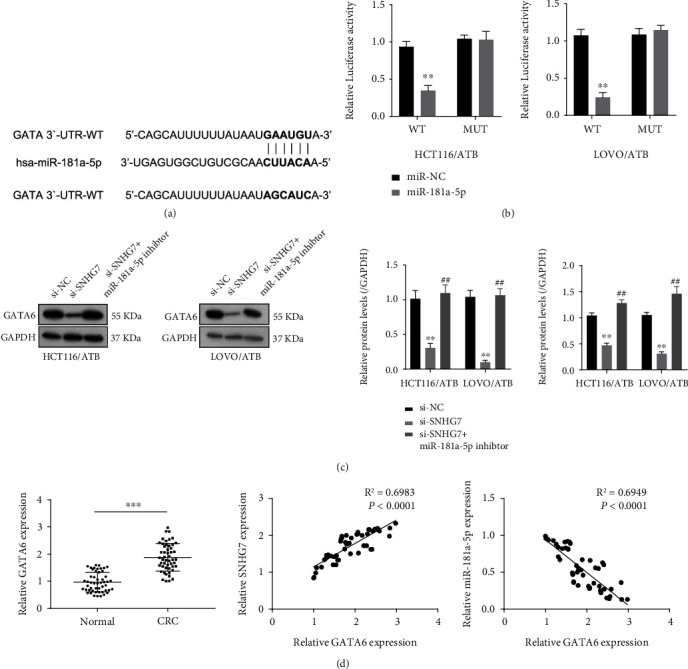
SNHG7 promotes GATA6 expression via sponging miR-181a-5p. (a) Potential binding site of miR-181a-5p in GATA6 found by TargetScan. (b) Luciferase reporter assay to verify the interaction between miR-181a-5p and GATA6 in HCT116/ATB and LOVO/ATB cell lines. miR-181a-5p overexpression inhibited Luciferase activity, but the inhibition disappeared when the binding site was mutated in GATA6. (c) Western blot and qRT-PCR showing the downregulation of GATA6 by SNHG7 knockdown and the upregulation of GATA6 by the transfection of miR-181a-5p in HCT116/ATB and LOVO/ATB cell lines. (d) qRT-PCR measurement to check the expression level of GATA6 in 50 pairs of CRCs and the adjacent tissues. A significant negative correlation between GATA6 and miR-181a-5p and a significant positive correlation between GATA6 and SNHG7 were observed by Spearman correlation analysis in 50 cases of CRC. The statistical differences were evaluated by Student's *t*-test (∗*P* < 0.05, ∗∗*P* < 0.01, and ∗∗∗*P* < 0.001).

We used TargetScan to screen the potential target gene regulated by miR-181a-5p and found that GATA6 contained a binding site of miR-181a-5p (Figure 4(a)). We employed the Luciferase reporter assay to verify the interaction between miR-181a-5p and GATA6. Overexpression of miR-181a-5p inhibited the Luciferase activity in HCT116/ATB and LOVO/ATB cell lines but did not in the cells with the miR-181a-5p binding site mutated (Figure 4(b)). Next, the relationship between SNHG7, miR-181a-5p, and GATA6 was explored. In CRC tissues, the expression level of GATA6 was higher in the CRC tissues than that in the healthy adjacent tissues. The expression level of GATA6 was positively correlated with SNHG7 while negatively with miR-181a-5p (Figure 4(d)). Furthermore, we investigated whether SNHG7 could regulate the expression of GATA6. In ATB-resistant cell lines, down-regulation of SNHG7 decreased the expression of GATA6, which was partially recovered by the addition of the miR-181a-5p inhibitor (Figure 4(c)). The above results demonstrate that SNHG7 regulates GATA6 expression via sponging miR-181a-5p. Furthermore, we detected the protein level of GATA6 using western blot in HCT116/ATB cells transfected with OE GATA6 (Figure S1B). The results presented that the expression level GATA6 increased significantly, indicating that the transfection of the overexpression construct was successful.

### 3.5. SNHG7 Regulates CRC Progression and ATB Resistance of CRC by Modulating GATA6 Expression via Sponging miR-181a-5p

**Figure 5:**
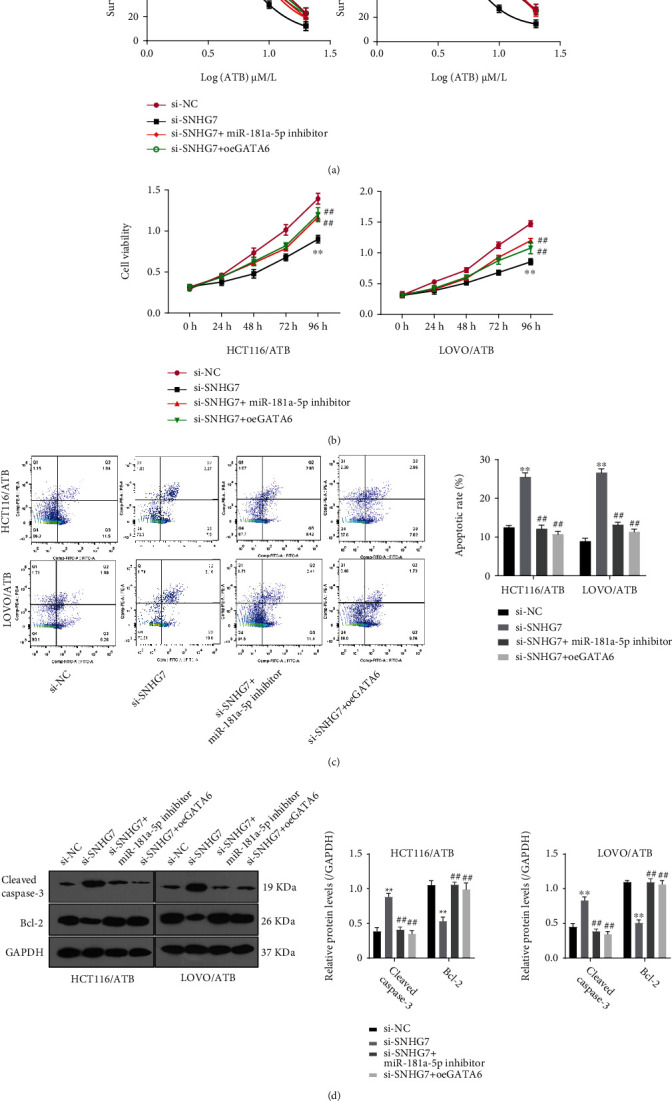
SNHG7 knockdown improves the ATB-sensitivity of CRC by regulating miR-181a-5p/GATA6. (a) Survival rate of HCT116/ATB and LOVO/ATB cell lines treated with 0, 2, 5, 10, and 20 *μ*M ATB. SNHG7 knockdown decreased the survival rate, which was partially recovered by overexpressing GATA6 or miR-181a-5p inhibitor. (b) Cell viability of siSNHG7 cell lines. The decreased cell viability was partially recovered by overexpressing GATA6 or miR-181a-5p inhibitor. (c) Flow cytometric analysis of the level of cell apoptosis. Down-regulation of SNHG7 in HCT116/ATB and LOVO/ATB cell lines increased cell apoptosis, which was partially reversed by overexpressing GATA6 or miR-181a-5p inhibitor. (d) Western blot to determine the levels of caspase-3 and Bcl-2, apoptosis-related proteins. Caspase-3 expression was increased, but Bcl-2 expression was decreased in siSNHG7 cell lines, which was partially reversed by overexpressing GATA6 or miR-181a-5p inhibitor. The statistical comparison was done by Student's *t*-test (∗*P* < 0.05, ∗∗*P* < 0.01, and ∗∗∗*P* < 0.001).

Having demonstrated that SNHG7, miR-181a-5p, and GATA6 jointly form a regulatory network in CRC, we set out to explore the role of the SNHG7/miR-181a-5p/GATA6 axis in the resistance to ATB. Compared with the control, SNHG7 knockdown decreased the survival rate of ATB-resistant CRC cells treated with different concentrations of ATB, which was partially recovered by overexpressing GATA6 or adding miR-181a-5p inhibitor (Figure 5(a)). In addition, GATA6 overexpression or miR-181a-5p inhibition could recover the cell proliferation ability of si-SNHG7 cell lines (Figure 5(b)). Flow cytometric analysis showed that GATA6 overexpression or miR-181a-5p inhibition enhanced the cell apoptosis of si-SNHG7 cell lines (Figure 5(c)). Correspondingly, the expression of caspase-3 was decreased, whereas the expression of Bcl-2 was increased in si-SNHG7 cells overexpressing GATA6 or with miR-181a-5p inhibited (Figure 5(d)). These results demonstrate that SNHG7/miR-181a-5p/GATA6 axis plays an important role in ATB resistance in CRC.

## 4. Discussion

ATB, a multikinase angiogenesis inhibitor, can effectively inhibit VEGFR, PDGFR, FGFR, c-kit, and other kinases, which could induce cell apoptosis and inhibit cell proliferation via the Erk and Akt pathway [37]. In 2018, it was approved to clinically treat locally advanced or metastatic non-small cell lung cancer by the National Medical Products Administration (GYZZ H20180002, GYZZ H20180003, and GYZZ H20180004). However, like the situation with many other targeted therapy drugs, drug resistance to ATB in multiple tumors has developed. Intrinsic or acquired resistance is a complicated and multifactorial event and remains a major challenge for the OS of patients with advanced CRC undergoing drug therapy. The improvement of anti-apoptotic ability, such as the expression of anti-apoptotic genes or the loss of pro-apoptotic genes, is an important mechanism of drug resistance in tumor cells [34, 35]. In the past decades, numerous dysregulated lncRNAs have been found to be associated with the carcinogenesis and progression of various malignancies [38]. SNGH7 has been reported to be upregulated in multiple tumors, including CRC, which enhances cell proliferation and inhibits apoptosis [10, 13–15], suggesting that SHNG7 may be involved in ATB resistance in CRC. In this study, we found that SNHG7 was significantly up-regulated in CRC and ATB-resistant CRC cell lines than that in the control. In addition, the high expression levels of SNHG7 were significantly related to the poor OS. Therefore, these results implicate that SNHG7 plays an important role in the resistance to ATB in CRC.

To further elucidate the role of SNHG7 in the resistance to ATB, SNHG7 expression was silenced by RNA interference in ATB-resistant cell lines HCT116 and LOVO cells. The cells with low expression of SNHG7 exhibited decreased cell proliferation and increased apoptosis. In addition, SNHG7 knockdown sensitized CRC cells to resist ATB. SNHG7 typically acts as a ceRNA to sponge various miRNA and regulate the expression of their target genes in different malignancies, including CRC. In this study, we found that miR-181a-5p is the direct target of SNHG7. Our results revealed a negative correlation between SNHG7 and miR-181a-5p. Furthermore, we found a positive correlation between SNHG7 and GATA6 in CRC. Overexpression of GATA6 or addition of miR-181a-5p inhibitor could partially reverse the effect of SNHG7 knockdown, such as decreased cell proliferation ability, increased apoptosis, and increased susceptibility to ATB. In summary, our results elucidate that SNHG7 upregulation contributes to the resistance to ATB in CRC by regulating the miR-181a-5p/GATA6 axis. Notably, SNHG7 has been reported to sponge other miRNAs and regulate the expression of relevant genes. Shan et al. [11] and Li et al. [15] reported that SNHG7 can regulate miR-216b/GALNT1 and miR-34a/GALNT7 to promote cell proliferation, migration, and invasion in CRC. As such, SNHG7 could regulate multiple miRNA/target gene axis to promote tumorigenesis and ATB resistance in CRC.

However, the pathways downstream of the SNHG7/miR-181a-5p/GATA6 axis still remain elusive. GATA6 can bind to the promotor of uPA to activate its expression [25]. In CRC, GATA6 overexpression could directly activate the expression of leucine-rich repeat-containing G protein-coupled receptor 5 and subsequent Wnt signaling pathway [39], as well as REG4 [40], thereby promoting the development and progression of CRC. GATA6 could also activate the Wnt signaling pathway by negatively regulating the Wnt antagonist Dickopf-1 to promote PC development [41]. Therefore, in the future, we will test whether the SNHG7/miR-181a-5p/GATA6 axis activates the Wnt signaling to promote cell proliferation and ATB resistance in CRC. In addition, Tankyrase inhibition acting on the Wnt/*β*-catenin pathway could recover resistance to PI3K and AKT inhibitors in the treatment of CRC [41]. ATB can induce cell apoptosis and inhibit cell proliferation via ERK and AKT pathways [37]. Therefore, it is reasonable to speculate that Tankyrase inhibition can restore the ATB resistance in CRC as well. In this context, whether SNHG7 could regulate the expression of Tankyrase merit a future study.

In conclusion, our study has revealed that SNHG7 was upregulated in the tissues and cell lines of CRC and ATB-resistant CRC cells. SNHG7 acts as a ceRNA to promote cell proliferation, anti-apoptosis ability, and ATB resistance by regulating the miR-181a-5p/GATA6 axis in CRC. Our findings provide a novel insight into the underlying molecular mechanism of SNHG7 in promoting ATB resistance in CRC.

## Data Availability

All supporting data of this work, which are not available in public because of ethical restrictions, are available from the corresponding author upon request.

## Supplementary Materials

Supplementary Materials
**Figure S1.** (A) Tumor sizes in xenograft models were measured every 7 days until 28 days after establishment. (B) Western blot detected the significantly increased expression level of GATA6 in HCT116/ATB cells transfected with OE GATA6.
