# Supplementary material for: Downregulation of LncRNA SNHG7 Sensitizes Colorectal Cancer Cells to Resist Anlotinib by Regulating miR-181a-5p/GATA6
Source: Gastroenterol Res Pract. 2023 Jan 14;2023:6973723. doi: 10.1155/2023/6973723 (PMC9867592; doi:10.1155/2023/6973723)
Supplement: Supplementary Materials — Figure S1. (A) Tumor sizes in xenograft models were measured every 7 days until 28 days after establishment. (B) Western blot detected the significantly increased expression level of GATA6 in HCT116/ATB cells transfected with OE GATA6. [file 6973723.f1.docx]

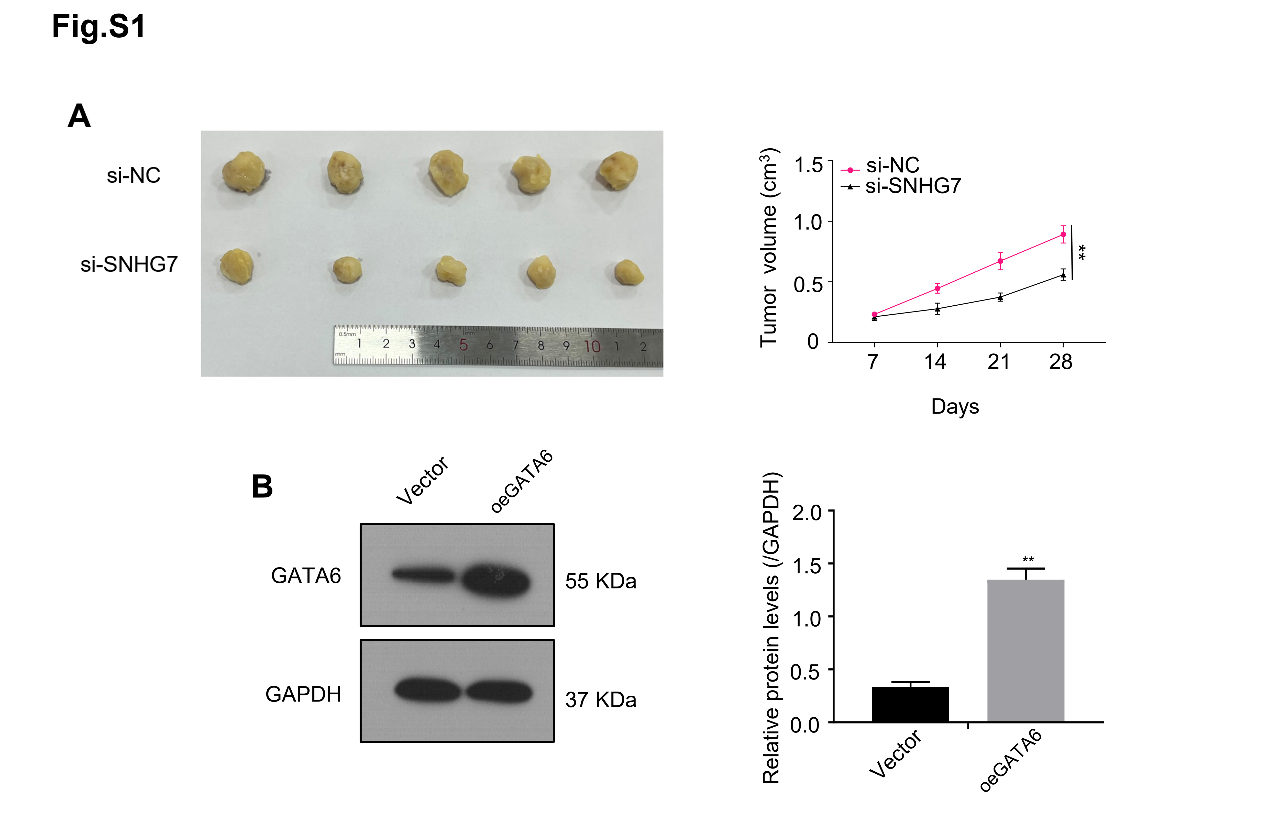


**Figure S1.** (A) Tumor sizes in xenograft models were measured every 7 days until 28 days after establishment.

(B) Western blot detected the significantly increased expression level of GATA6 in HCT116/ATB cells transfected with OE GATA6.
